# Supplementary material for: Neutral Sphingomyelinase 2 Inhibition Limits Hepatic Steatosis and Inflammation
Source: Cells. 2024 Mar 6;13(5):463. doi: 10.3390/cells13050463 (PMC10931069; doi:10.3390/cells13050463)
Supplement: Supplementary file 1 [file cells-13-00463-s001.zip › cells-2864141-supplementary.pdf]

Supplementary Figure S1. Quantification of ALT and AST levels in mice plasma

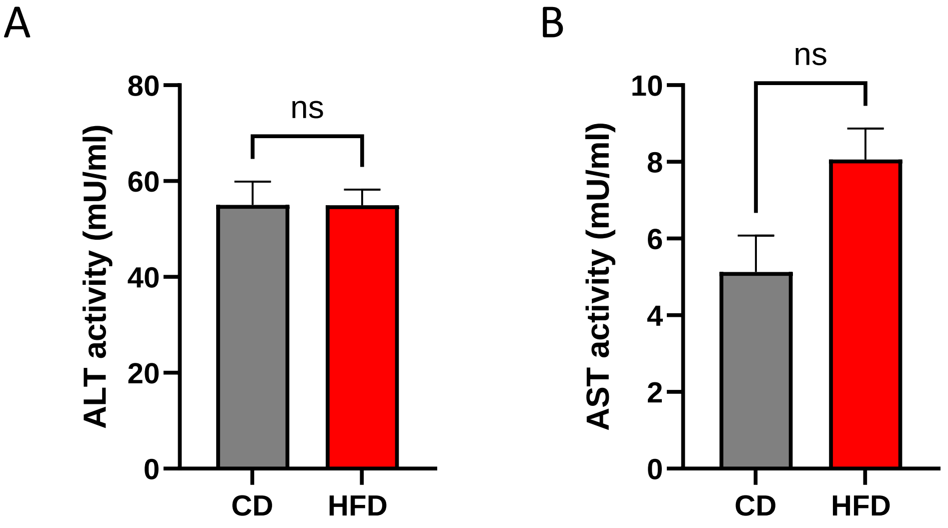

Supplementary Figure S1**.** Quantification of ALT and AST levels in plasma of mice fed with a diet-induced steatosis. The plasma levels of ALT were measured using the Alanine Transaminase Activity Assay Kit (Abcam, ab-105134), according to the manufacturer's instructions. The plasma levels of AST were measured using the Aspartate Aminotransferase Activity Assay Kit (Abcam, ab-105135), according to the manufacturer's instructions.

(A) ALT activity **(B)** AST activity Results were obtained from a minimum of n=5**.** All data are expressed as mean ± SEM .

Supplementary Figure S2 . Increase of nSMase gene expression livers of mice fed with a diet-induced steatosis

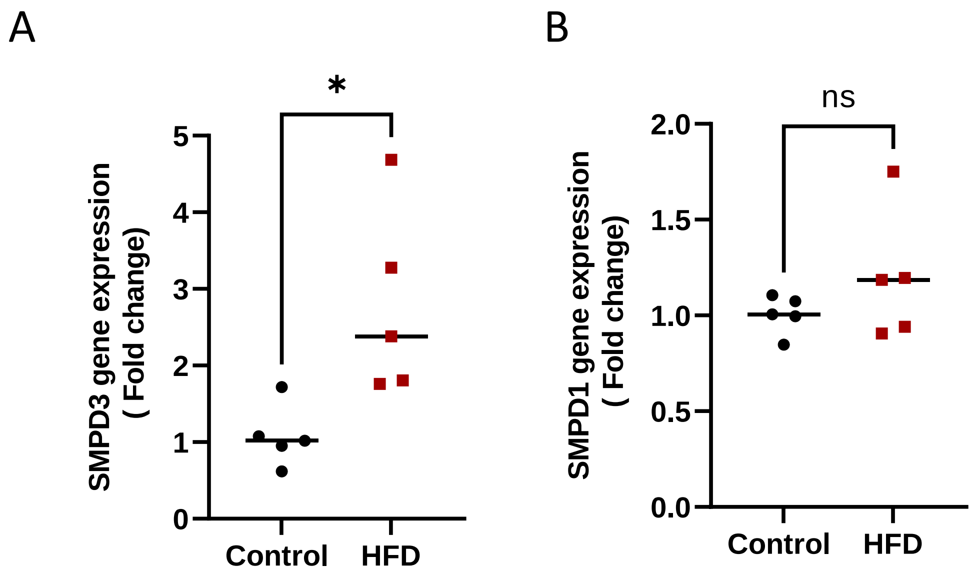

Supplementary Figure S2**.** Increase of nSMase gene expression livers of mice fed with a diet-induced steatosis. **(A)** qRT-PCR analysis of SMPD3 gene expression. **(B)** qRT-PCR analysis of SMPD1 gene expression. Results were obtained from a minimum of n=5. All data are expressed as mean ± SEM . * p ≤ 0.5,** p ≤ 0.1 *** p ≤ 0.001, **** p ≤ 0.0001 and ns: non-significant. Images are shown at 20 magnification; Scale bar = 50 m.

Supplementary Figure S3. Inducing hepatic steatosis with high glucose and high oleate acid culture in HepG2

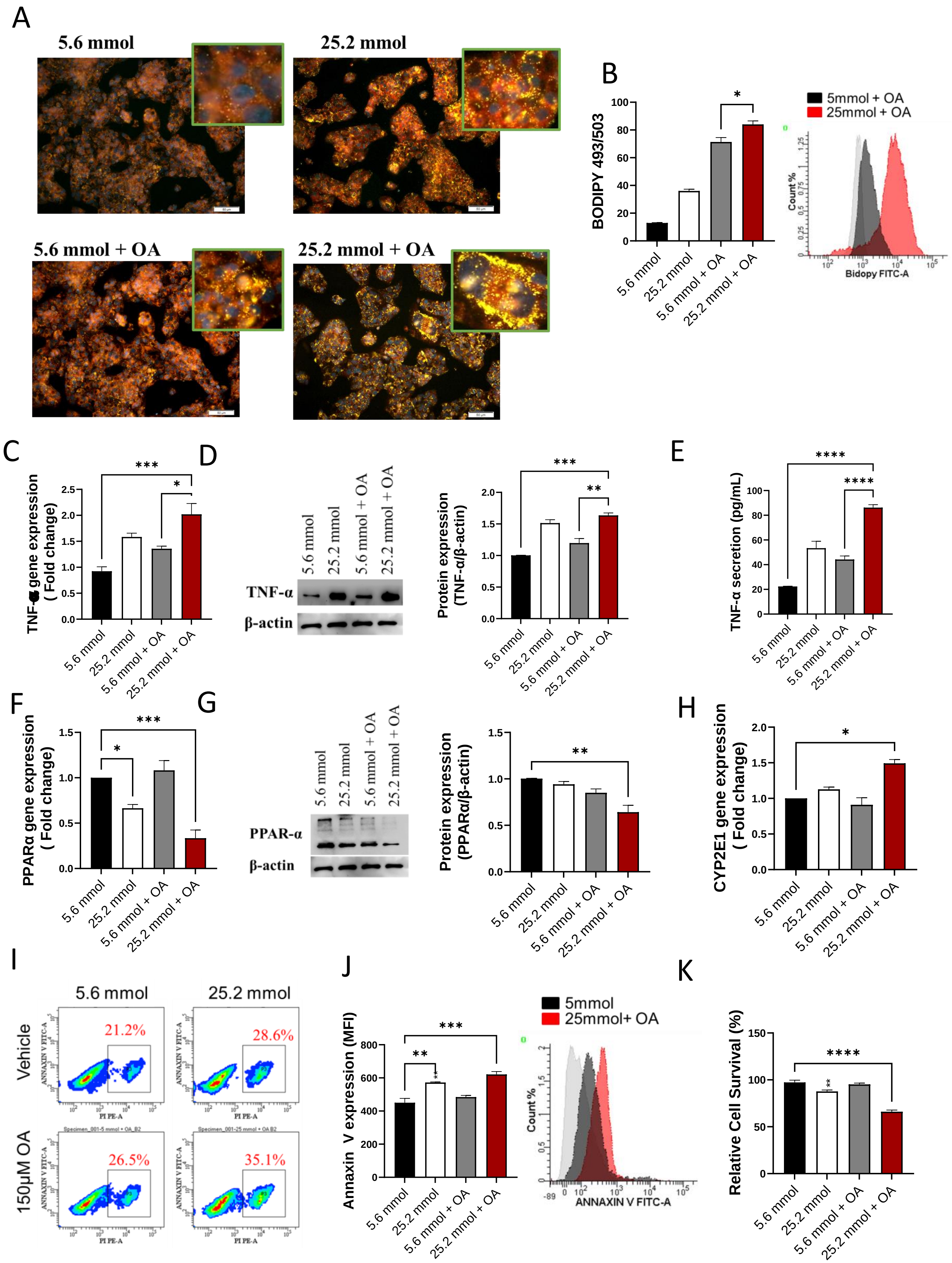

Figure S3. Inducing hepatic steatosis with high glucose and high oleate acid culture in HepG2. HepG2 cells were cultured in either low (5.6 mmol) D-glucose or high (25.2 mmol) D-glucose in the presence or absence of OA stimulation. (A) Nile red fluorescence staining; (B) Bar graph of median fluorescence intensity (MFI) of BODIPY 493/503 calculated from three independent determinations, with similar results presented in the histogram; (C) qRT-PCR analysis of TNF-α expression; (D) TNF-α protein analysis with representative immunoblot; (E) TNF-α protein secretion in the media measured by enzyme-linked immunosorbent assay; (F) qRT-PCR analysis of PPARα expression; (G) PPARα protein analysis with representative immunoblot; (H) qRT-PCR gene expression analysis of Cyp2E1 expression; (I) Representative flow cytometry plots using Annexin V-FITC/PI staining for apoptosis; (J) Bar graph of MFI of Annexin V-FITC calculated from three independent determinations, with similar results presented in the histogram; (K) Bar graph of viable cells percentage analyzed using an MTT assay. Results were obtained from a minimum of three independent experiments. All data are expressed as mean ± SEM. * $p \leq 0.5$, ** $p \leq 0.1$ *** $p \leq 0.001$, **** $p \leq 0.0001$ and ns: non-significant. Images are shown in 20x magnifications: Scale bar = 50 m.

Supplementary Figure S4: Effect of nSMase inhibition in HepG2 liver steatosis model

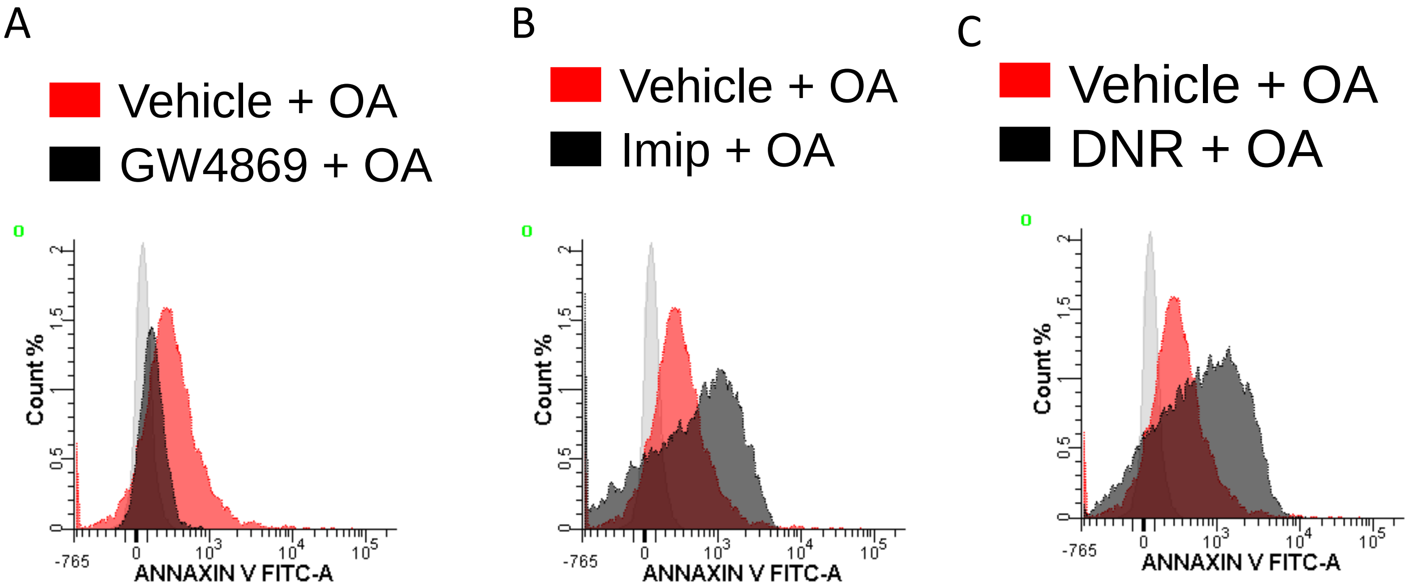

Figure S4. Effect of nSMase inhibition in HepG2 liver steatosis model . Represented histograms of fluorescence intensity (MFI) of BODIPY 493/503 in **(A)** Under GW4869 inhibition . **(B)** Under Imip inhibition and **(C)** Under DNR inhibition .
